# GRAVEN: a database of teaching method that applies gestures to represent the neurosurgical approach’s blood vessels and nerves

**DOI:** 10.1186/s12909-024-05512-0

**Published:** 2024-05-07

**Authors:** Hanwen Xuan, Junzhe Zhong, Xinyu Wang, Yu Song, Ruofei Shen, Yuxiang Liu, Sijia Zhang, Jinquan Cai, Meichen Liu

**Affiliations:** 1 Department of Neurosurgery, Neuroscience Institute, the Second Affiliated Hospital of Harbin Medical University, Heilongjiang Academy of Medical Sciences, Harbin, 150086 China; 2 Department of Modern Education Technology Center, Harbin Medical University, Harbin, 150086 China; 3 Department of Educational Administration, the Second Affiliated Hospital of Harbin Medical University, Harbin, 150086 China; 4 Future Medical Laboratory, The Second Affiliated Hospital of Harbin Medical University, Harbin, 150086 China

**Keywords:** Neurosurgical education, Gesture, Intracranial anatomy, Database

## Abstract

**Background:**

In this era of rapid technological development, medical schools have had to use modern technology to enhance traditional teaching. Online teaching was preferred by many medical schools. However due to the complexity of intracranial anatomy, it was challenging for the students to study this part online, and the students were likely to be tired of neurosurgery, which is disadvantageous to the development of neurosurgery. Therefore, we developed this database to help students learn better neuroanatomy.

**Main body:**

The data were sourced from *Rhoton’s Cranial Anatomy and Surgical Approaches* and *Neurosurgery Tricks of the Trade* in this database. Then we designed many hand gesture figures connected with the atlas of anatomy. Our database was divided into three parts: intracranial arteries, intracranial veins, and neurosurgery approaches. Each section below contains an atlas of anatomy, and gestures represent vessels and nerves. Pictures of hand gestures and atlas of anatomy are available to view on GRAVEN (www.graven.cn) without restrictions for all teachers and students. We recruited 50 undergraduate students and randomly divided them into two groups: using traditional teaching methods or GRAVEN database combined with above traditional teaching methods. Results revealed a significant improvement in academic performance in using GRAVEN database combined with traditional teaching methods compared to the traditional teaching methods.

**Conclusion:**

This database was vital to help students learn about intracranial anatomy and neurosurgical approaches. Gesture teaching can effectively simulate the relationship between human organs and tissues through the flexibility of hands and fingers, improving anatomy interest and education.

## Background

In this era of rapid development of information technology, many medical school teachers have recorded online courses to help students better learn relevant knowledge [1]. However, the effect of online teaching is often poor, which makes it difficult for medical students to learn and remember the relevant knowledge of anatomy, not to mention the more difficult neuroanatomy of anatomy. The shift from offline to online teaching of anatomy inevitably led to some confusion [2].

Of all surgical procedures, neurosurgery is often more difficult. Neurosurgery’s difficulty mainly lies in identifying complex anatomical structures near the lesion site [3]. Complex intracranial anatomy is also daunting for undergraduates and neurosurgery residents. In addition, neurosurgeons have more years of training than any other surgeons [4]. Part of the reason that neurosurgeons take too long to train is also due to the complex anatomy of the brain. An excellent foundation in intracranial anatomy is essential for a neurosurgeon.

In the training process of undergraduates, the anatomy of the nervous system is complicated, and the typical teaching methods are still classroom slides, autopsies, etc.; The scheduling of courses related to the nervous system has different degrees of fragmentation, and there are significant differences in learning efficiency and interest among students [5].

In the postgraduate stage, after entering the clinical work of neurosurgery, because of different craniotomies and various microinvasive localizations of the lesions under the complex anatomic structure, it is difficult for most students to learn and identify intracranial vascular and nerve deformities. Graduate students and teachers are trapped in clinical practice and need to pay more attention to the importance of primary anatomy education. The fundamental reason is that there needs to be an effective teaching method for neurosurgery that can help graduate students systematically study standard surgical approaches.

Neurosurgeons and bystanders, faced with a narrow field of view (2–3 cm in diameter of the bone window in a “Keyhole” craniotomy) and different patient postures, may lose important anatomical landmarks that guide the surgical approach during surgery; therefore, it is essential to trace the origin of a blood vessel according to its direction of flow. In addition, accurately identifying the distribution and deformation of the nerve and blood vessels is an important method to determine the operative clearance and level in neurosurgery. It is also the decisive basis for the precise treatment of lesions and the reduction of surgical damage to normal tissues. At present, there is a lack of teaching methods in the neurosurgical approach.

With the development of science and technology, technologies such as virtual reality (VR) [6]and 3D printing [7] are gradually being applied to neurosurgery teaching, which is very important for neurosurgery education. Nevertheless, the expensive cost of equipment makes it difficult for many medical schools to achieve the popularization of these technologies. Therefore, the current teaching methods of mainstream medical schools are still traditional. However, traditional teaching methods cannot vividly show the intracranial anatomical structures, which makes it difficult for students to understand many vital structures in the brain and leads to students’ sense of frustration; in turn, it is challenging to develop an interest in neurosurgery, which is disadvantageous to neurosurgery education [8]. Currently, most medical education in neurosurgery has little connection with the surgical approach. Neurosurgery advocates a minimally invasive method to reduce the injury of craniotomy. As much as possible, a small bone window should be used to expose large intracranial lesions, and the flow direction and adjacent area of the blood vessels in the small operative field should be the teaching focus. Neuroanatomy is the foundation of neurosurgeons and affects the judgment of complicated conditions during surgery. Therefore, the core of neurosurgery teaching should be to speed up the growth process of medical students from “Understanding neuroanatomy” to “Understanding the surgical approach.” In this research, we established a database that applies Gestures to Represent the neurosurgical Approach’s blood Vessels and Nerves (GRAVEN: www.graven.cn). Using this database can quickly help students and neurosurgery residents to build an interest in neurosurgery and study intracranial anatomy.

## Construction and content

The data used to generate this online database were taken from *Rhoton’s Cranial Anatomy and Surgical Approaches* [9] and *Neurosurgery Tricks of the Trade* [10]. We designed corresponding hand gesture figures for each atlas of anatomy and marked the legend and direction of blood flow on the two types of figures.

**Fig. 1:**
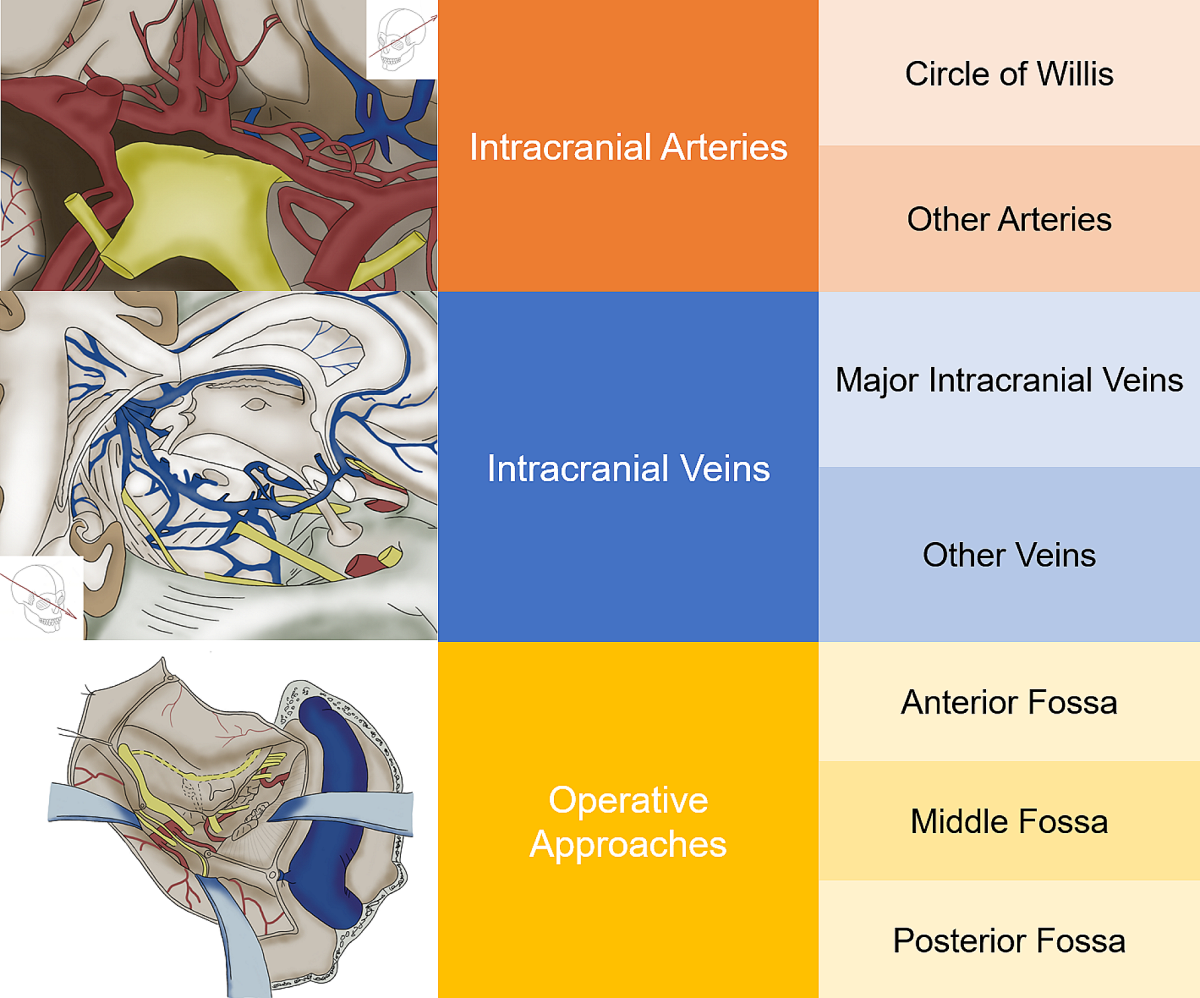
This is the directory of contents that the database contains

The database contains three parts: the anatomy of important intracranial arteries, important intracranial veins, and neurosurgical approaches, where we indicate the direction of flow of blood vessels by pointing an arrow, the finger represents the blood vessels and nerves, and the relative position between the fingers defines the relative position between the blood vessels/nerves (Fig. 1).

We apply hand gestures to represent the major blood vessels, such as the Internal Carotid Artery, Basilar Artery, Superior Cerebellar Artery. These vessels are often the first lesson in the anatomy of intracranial vessels. Using hand gestures can help students understand the essential major blood vessels in the brain more quickly and conveniently, help students form a general impression of blood vessel movement and distribution in the brain, and lay a foundation for the subsequent explanation of the anatomy of the surgical approach. The application of hand gestures to indicate blood vessels has many advantages: to help students understand the flow of blood vessels, remember the branches of blood vessels, and understand the blood supply area.

**Fig. 2:**
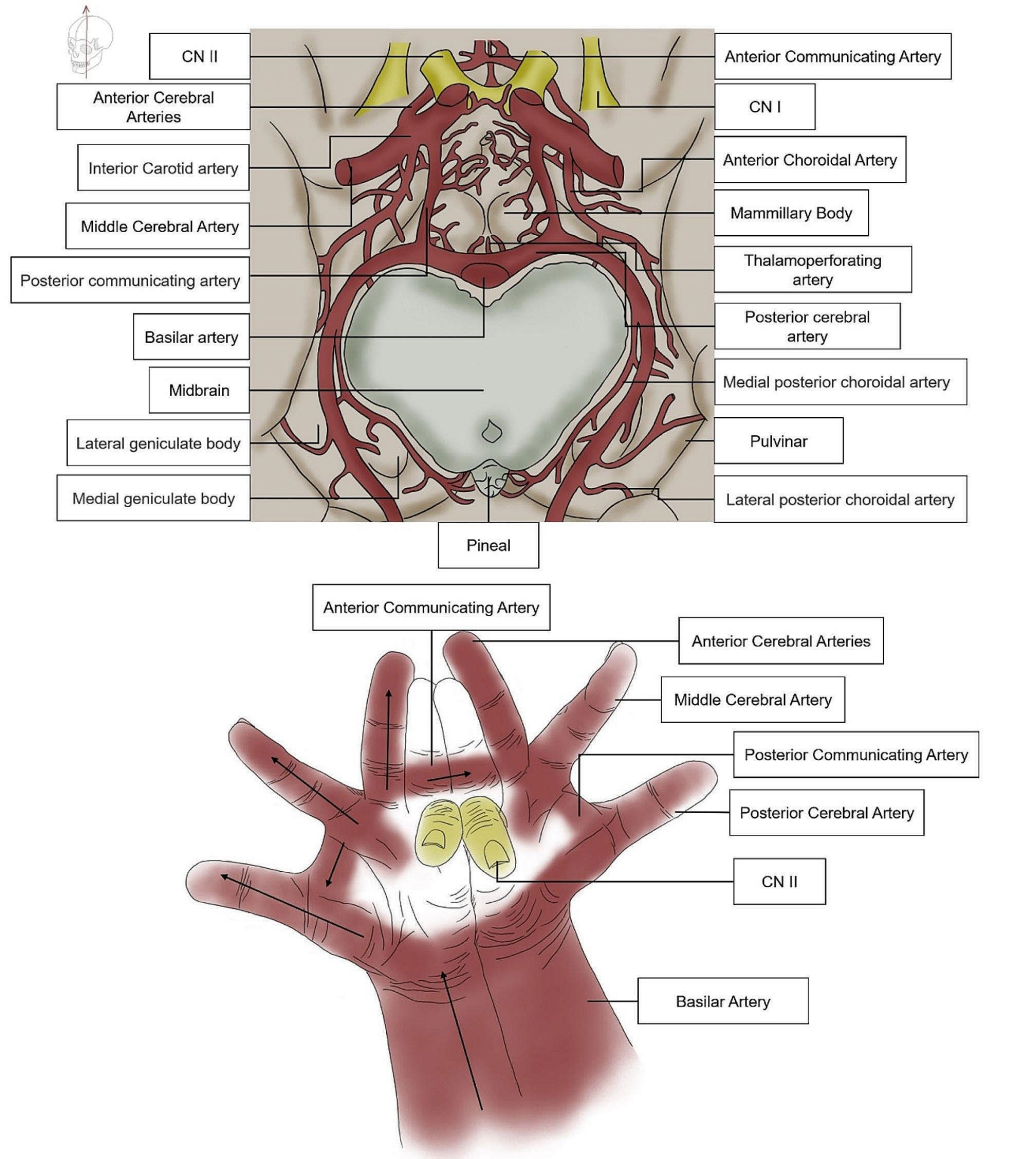
The circle of Willis and its gesture

For example, the circle of Willis is one of the most important arteries in the brain. Its function is that, under normal circumstances, blood on both sides of the circle of cerebral arteries does not mix but instead serves as a potential device for compensation; when an artery is stunted or blocked, it can be regulated to some extent by the circle of Willis to redistribute and compensate the blood and maintain the nutrition and function of the brain. We apply gestures to vividly represent the circle of Willis (Fig. 2). We apply the wrist to represent the Interior Carotid Artery, the thumb to represent the Posterior Communicating Artery, the index finger to represent the Middle Cerebral Artery, the middle finger to represent the Anterior Cerebral Artery, the ring finger to represent the Anterior Communicating Artery, the little finger to represent the Olfactory Nerve.

Applying gestures to represent the circle of Willis can help students understand the movement and distribution of blood vessels more clearly, and deepen their memory of the circle of Willis.

In neurosurgery, we should recognize the essential arteries and learn to recognize the crucial intracranial veins, such as the Vein of Galen, and Internal Cerebral Vein. Using gestures, we can create a stereogram of the flat blood vessels in the book, thus deepening the students’ memory of it.

**Fig. 3:**
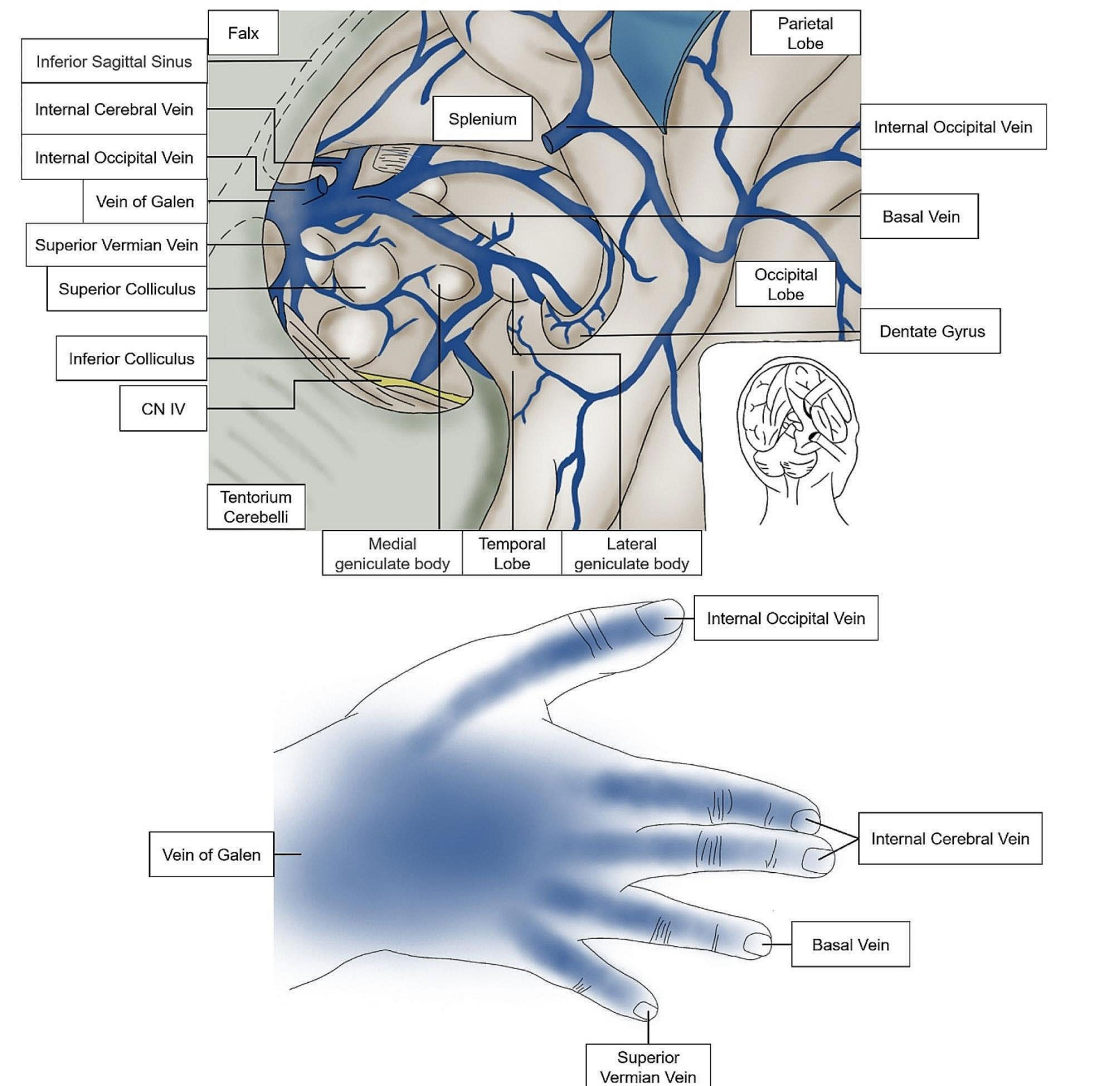
The branches of the Vein of Galen and its gesture

For example, the memory and learning of the Vein of Galen and its branches often require more clarification for students (Fig. 3). We apply the dorsum of the hand to represent the Vein of Galen, the thumb to represent the Internal Occipital Vein, the index finger and middle finger to represent the Internal Cerebral Vein, the ring finger to represent the Basal Vein, and the little finger to represent the Superior Vermian Vein. Showing the cerebral veins and their branches through hand gestures allows students to learn better and faster about the contents of these veins.

Because of the particularity of brain anatomy, there is no universal approach to any part of the brain, leading to many classical surgical approaches. These classic surgical approaches are complex for students who are new to neurosurgery. By using hand gestures, we describe the proximity of essential blood vessels and nerve structures in the surgical approach with the relative position between the fingers, thereby deepening the students’ memory of the anatomy of the structures in the surgical approach and helping students understand its structure. Regarding the neurosurgical approach, we divided the approach into three parts: anterior fossa, middle fossa, and posterior fossa.

**Fig. 4:**
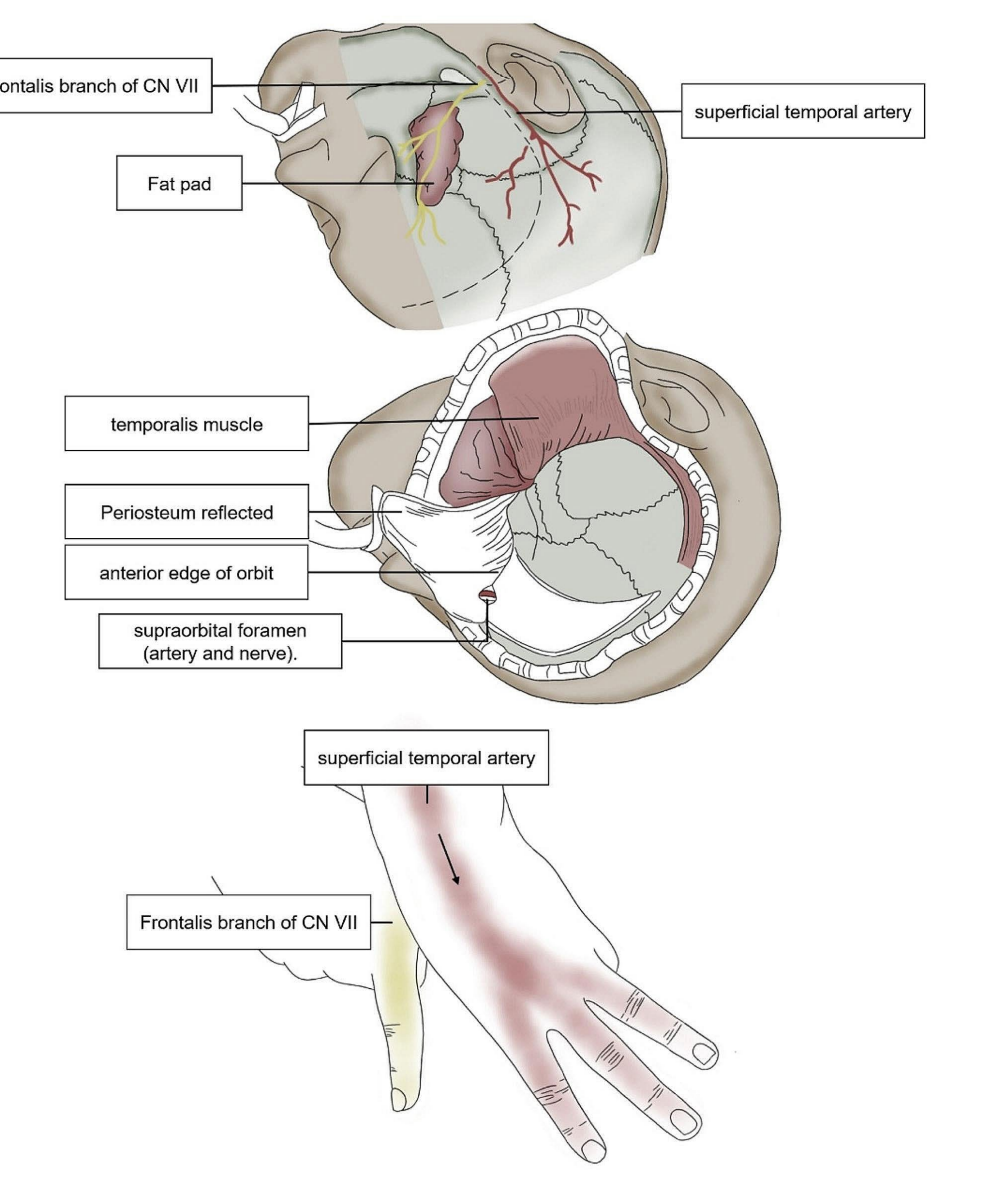
The pterional approach and its gesture

For example, the pterional approach is one of the most commonly used approaches in neurosurgery. We use hand gestures to represent the common blood vessels and nerves in the pterional approach (Fig. 4), which makes it easy for students to learn and remember the pterional approach and lets students quickly get started on the critical structures of the pterional approach, thus breaking the students’ prejudices about the difficulty of getting started in neurosurgery.

We used Power Analysis and Sample Size (PASS) software to calculate the sample size [11]. Briefly, the sample size was 38, with a type I error rate of 0.05, power 85%, and two-sided test. Assuming a drop-out rate of 20%, a total of 48 students were needed. We recruited medical undergraduates in this study from January 1st, 2024- January 31st, 2024. A total of 50 students were randomized to two different groups of studying Neuroanatomy. The results of the Age were used t-test, and the p-value is non-significant (*p* = 0.42). Using Chi-square test for gender difference between two groups, and the p-value is non-significant (*p* = 0.77) (Table 1). Randomization was performed through online-based random list generator services (Randomness and Integrity Services Ltd., Dublin, Ireland). All recruited participants completed the study and no data was withdrawn. Neuroanatomy instruction was performed with traditional teaching methods (Power Point Teaching, *n* = 25) based on textbooks Rhoton’s Cranial Anatomy and Surgical Approaches and Neurosurgery Tricks of the Trade or GRAVEN database combined with above traditional teaching methods(*n* = 25). The requirement for participants was the completion of a 25-question neuroanatomy test to evaluate acquired knowledge developed by two neurosurgeons at the end of class. The text used a percentage grading system. The pass mark was set at 60 per cent. Results revealed a significant improvement (*p* = 0.0026) in exam grading in using GRAVEN database combined with traditional teaching methods (Mean = 78.84, SD = 4.78) compared to the traditional teaching methods (Mean = 73.80, SD = 6.32). The results of the exam grading were used t-test (Table 2). Survey data of questionnaire at the end of class was collected from all 25 students. Students were asked to answer the questions using a 5-point Likert scale (1= “strongly disagree”, 2= “disagree”, 3= “neutral,” 4= “agree”, to 5 = “strongly agree”) after class. In the question of “GRAVEN were an interesting teaching method”, 11 students chose “agree” and 10 students chose “strongly agree”, which revealed twenty-one (84%) students felt GRAVEN database combined with traditional teaching enhanced the interest in neuroanatomy. And in the question of “GRAVEN were helpful in learning neuroanatomy”, 7 students chose “agree” and 10 students chose “strongly agree”, which revealed seventeen (68%) students felt GRAVEN database combined with traditional teaching more helped them study neuroanatomy (Table 3).

**Table 1:** Outline of participant demographics

Characteristics	GRAVEN(*N* = 25)	Traditional teaching(*N* = 25)	Total (*N* = 50)	*p*
Age, mean(± SD)	18.8(± 0.81)	19(± 0.95)	18.9(± 0.88)	0.42
Male, *n* (%)	11(44%)	10(40%)	21(42%)	0.77
Female, *n* (%)	14(56%)	15(60%)	29(58%)	

**Table 2:** Overview of student exam grading

	GRAVEN (mean ± SD)	Traditional teaching (mean ± SD)	*p*
Exam Grading	78.84 ± 4.78	73.80 ± 6.32	*p* = 0.0026

**Table 3:** Results of Likert survey data obtained after class

Question	Average score
GRAVEN were helpful in learning neuroanatomy	4.08
GRAVEN were an interesting teaching method	4.24

## Utility and discussion

Yohannan and colleagues [12]developed a new tool for using hand gestures to help students learning the spatial anatomy, which has been named as” Air Anatomy”. In addition, using a randomized controlled trial design, this study explored the use of a unique combination of hand gestures to enhance spatial anatomical understanding. The study suggested that “Air Anatomy” was a useful, “no-cost”, accessible method that aids spatial understanding of anatomical concept. Hill and colleagues [13] described two gesture-based techniques aimed at simplifying the anatomy of two complex intracranial nervous structures: the trigeminal nerve and the cerebral fornix. In our present work, we used hand gestures to describe the adjacency of neurosurgical anatomy structures and vascular flow direction of the surgical field and explore the role of enlightenment teaching mode with hand gestures as the core on undergraduate and postgraduate college training.

Our database is much more helpful for students to understand, learn, and remember intracranial anatomy and surgical approaches than the traditional book of anatomical drawings. Anatomy is a fundamental subject in the field of medicine. The content of neuroanatomy is complicated, which is the first impression of the learners. Through the flexibility of both hands and fingers, gesture teaching can effectively simulate the relationship between human organs and tissues and improve the interest in anatomy and teaching effect. Based on keeping the atlas of anatomy, gesture teaching changes the input of text and pictures into the visual output that can be executed and thought, which arouses the students’ subjective initiative and breaks through the traditional teaching material frame. The starting point of the gesture teaching method is the most familiar Atlas of anatomy. The endpoint is that the tissues of different lesion sites are adjacent to each other during the operation, which can prompt the students to start from the familiar details of the surrounding scenes, to study and think about the organization and structure of vascular deformation and nerve starting and stopping, and then to simulate with both hands, to naturally understand and master the different vascular deformation and flow direction in the process of application. This can cultivate students’ habit of active observation-summarization-application when they are faced with the situation in different approaches in the future. Further development of GRAVEN is foreseen in the near future to upload craniocerebral MRI or CT scan imaging. At present, by asking patients to make specific gestures, surgeon can protect brain functional areas of patients during the awake craniotomy [14]. In addition, gesture recognition technology also can standardize detection of surgeons’ gestures during surgery [15, 16]. In the future gestural instruction, we can bring gesture detection technology into the classroom, teachers can use the gesture detection technology in front of students to detect whether students’ gestures are standard during the teaching process, and can collect students’ gestures for homework to test students’ memory of the content of this lesson.

On the one hand, 3D models can achieve complex geometric shapes, providing higher design freedom; it can quickly produce prototypes, reducing development cycle and cost; it can meet customized production needs, providing flexibility for personal and small-scale production [17]. On the other hand, it may not meet the demand for rapid delivery in mass production; the types of materials available for 3D printing are relatively limited, especially in education field [18]. Virtual reality technology is a very novel teaching technology at present [19]. Students in the classroom can use virtual reality technology to learn complex cranial anatomy structures more intuitively and clearly [20]. However, the high price limit makes virtual reality technology not adopted in most medical colleges. Gesture teaching can make the otherwise obscure cranial anatomy lively and interesting [21], which is very important to improve students’ motivation to learn cranial anatomy.

Through gesture teaching, students can understand the structure and function of the brain more intuitively and better grasp the relevant knowledge. Compared with traditional lecture-based teaching, gesture teaching is more interactive and participatory, which can stimulate students’ interest and make them more actively engaged in learning. In addition, gesture teaching can also develop students’ practical skills [22]. Through hands-on experience, students can better understand the details and points of cranial anatomy, and improve their practical ability and operational skills. It is also very beneficial for students’ future career development. If traditional teaching can be combined with gesture teaching, it can not only improve students’ interest and enthusiasm in learning, but also cultivate their practical ability.

### Limitations

This study has limitations, as do all studies. Our database can be used by students studying neuroanatomy and neurosurgery residents. This study only counted the usage of this database by undergraduate students, and did not include neurosurgery residents. In addition, in order to focus on more clinically applications, clinical data such as CT or MRI scan imagines should be added to the database to further assist students or residents in learning neuroanatomy and the anatomy of neurosurgical approaches.

## Conclusions

We have developed a database of intracranial vessels and nerves represented by hand gestures for neuroanatomy instruction. Teachers can use this database to simplify complex brain structures in the course of online teaching, and it can also be used as a supplement to teaching resources after class. Pictures of hand gestures and atlas of anatomy are available to view on GRAVEN (www.graven.cn) without restrictions for all teachers and students.

## Data Availability

The data that support the findings of this study are openly available at www.graven.cn.

## Abbreviations

VR: Virtual reality
3D printing: Three-dimensional printing
MRI: Magnetic resonance imaging
CT: Computed tomography
